# Highly efficient CRISPR/Cas9 system in *Plasmodium falciparum* using Cas9-expressing parasites and a linear donor template

**DOI:** 10.1038/s41598-021-97984-z

**Published:** 2021-09-16

**Authors:** Tsubasa Nishi, Naoaki Shinzawa, Masao Yuda, Shiroh Iwanaga

**Affiliations:** 1 Laboratory of Medical Zoology, Department of Medicine, Mie University, Mie, Japan; 2 Department of Environmental Parasitology, Graduate School of Medical and Dental Sciences, Tokyo Medical and Dental University, Tokyo, Japan; 3 Department of Molecular Protozoology, Research Institute for Microbial Diseases, Osaka University, Osaka, Japan

**Keywords:** Parasite genetics, Parasite genomics

## Abstract

The CRISPR/Cas9 system is a powerful genetic engineering technology for *Plasmodium falciparum*. We here report further improvement of the CRISPR/Cas9 system by combining the Cas9-expressing parasite with a liner donor template DNA. The Cas9-expressing parasite was generated by inserting the *cas9* gene in the genome by double crossover recombination. The site-directed mutagenesis and the fusion of fluorescence protein was achieved within two weeks with high efficiency (> 85%), by transfecting the schizonts of the Cas9-expressing parasite with the liner donor template and the plasmid carrying the sgRNAs. Notably, there were neither off-target mutations in the resultant transgenic parasites nor unexpected recombination, that are the technical problems of the current CRISPR/Cas9 system. Furthermore, with our system, two genes on different chromosomes were successfully modified in single transfection. Because of its high efficiency and robustness, our improved CRISPR/Cas9 system will become a standard technique for genetic engineering of *P. falciparum*, which dramatically advances future studies of this parasite.

## Introduction

Malaria is still a global public health threat, and its burden exceeds 200 million infections every year, resulting in more than 400,000 deaths annually^1^. *Plasmodium falciparum* is the most lethal human parasite among the five human malaria parasites and is responsible for most of those deaths. There is not yet an effective vaccine against *P. falciparum*, and the drug resistance against artemisinin, which is the current first line drug, has already emerged in endemic areas. Thus, countermeasures against infection are urgently required.

Genetic engineering of this parasite is an essential technology for investigating the function of genes, allowing the exploration of drug targets and vaccine antigens. The CRISPR/Cas9 system is a powerful genome editing technique employed in various living organisms and is now widely used in *Plasmodium* parasites^2–5^. In the parasites, the gene is modified through two steps as follows: the targeted genomic locus is cleaved by the Cas9-single guide RNA (sgRNA) complex, and the induced double-strand break is then repaired by homology-directed recombination (HDR) using donor template DNA. Several methods based on the CRISPR/Cas9 system have been developed in *P. falciparum* so far. First, the method using two plasmids coding the Cas9, the sgRNA, and the donor template DNA was developed^2,3^. Although this method was simpler and more efficient compared to the past genetic engineering methods^6,7^, it requires two kinds of drugs to select transgenic parasites, which decreases their growth rate and thus delays the establishment of the transgenic parasites. This technical limitation has been solved by the several methods using a single selectable marker. For example, the method using the suicide plasmid encoding Cas9 and sgRNA with the drug selectable marker together with the rescue plasmid carrying the donor template DNA without selectable marker was developed^8^. In addition, the method using the plasmid carrying all those three components was reported^9^. Furthermore, the method in which the Cas9-expressing parasites are transfected with the plasmid coding both the sgRNA and the donor template DNA were developed^10^. Although these methods succeeded to reduce the number of drug selectable markers used for genetic engineering, there is another technical problem that should be noted: unexpected recombination between the circular plasmid carrying the donor template DNA and the target locus^11–14^. This problem can be simply overcome by linearizing the donor template DNA, and in fact, some studies have reported genetic engineering by the CRIPSR/Cas9 systems using a linearized donor template DNA^2,15,16^. Briefly, a linear donor template and a plasmid having the Cas9/sgRNA were directly introduced into purified schizonts, which are RBC-invasive form of parasites, and then transgenic parasites were generated. However, since the linear DNA fragment is quickly lost from the parasites during nuclear division^7,15^, obtaining the transgenic parasite will be inefficient or even impossible if HDR between the cleaved site and the linear donor template do not occur immediately after transfection. Collectively, the technical limitation and the problems of CRISPR/Cas9 system of *P. falciparum* have not been fully solved, and further improvement is needed.

In our previous study, we successfully improved the CRISPR/Cas9 system in the rodent malaria parasite *P. berghei* by using a Cas9-expressing parasite and linear donor templates^13^. The Cas9-expressing parasites were co-transfected with a linear donor template and a plasmid carrying the sgRNA, achieving almost 100% genetic modification efficiency. Furthermore, any unexpected recombination did not occur in the resultant transgenic parasites. This suggests that to solve the technical problem and limitation associated with the current CRIPSR/Cas9 system, it might be important to use the Cas9-expressing parasite and a linear template DNA in combination. Based on this idea, we attempted to improve the CRISPR/Cas9 system in *P. falciparum* in this study. We first evaluated the efficiency of the transfection method using fully mature schizonts quantitatively, then confirmed whether it was sufficient for our proposed method. Next, we generated the Cas9-expressing parasites by incorporating the *cas9* gene into the genome by double-crossover recombination, and then engineered the genes by transfecting the purified schizonts of Cas9-expressing parasites with the linear donor template DNA and the plasmid carrying the sgRNA. In results, genes were engineered with high efficiency, approximately 85–100%, and there was no unexpected recombination in the resultant transgenic parasites. Furthermore, we were able to engineer two genes on different chromosomes simultaneously by using two linear donor templates and a plasmid with two sgRNAs, showing the applicability of our system. This improved system solved the current technical problems of the CRISPR/Cas9 system in *P. falciparum* and will thus be the standard method for genetic engineering of this parasite.

## Results

### Development of a DNA transfer method using fully mature schizonts of *P. falciparum*

**Figure 1:**
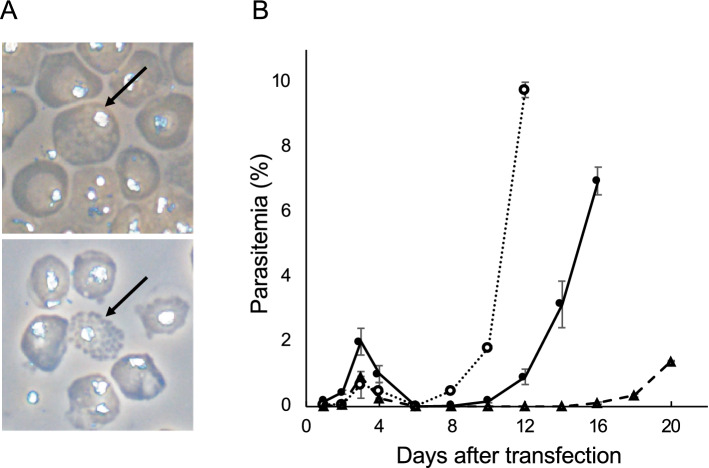
Transfection of fully mature schizonts of *P. falciparum*. (**A**) The fully mature schizonts enclosed within a single membrane of the parasitophorous vacuole (upper) or erythrocyte (lower) are indicated by arrows. (**B**). Purified schizonts, including fully mature schizonts, were transfected with 5 μg (closed circle/solid line) and 50 μg (opened circle/dashed line) of pFCENv1 plasmid. Five micrograms of pFCENv1 was also preloaded into RBCs and then introduced into the parasite by uptake (closed triangle/dashed line).

Full mature schizonts are known to be the developmental stage of *Plasmodium* parasites suitable for DNA transfer. Transfection method using the parasites at this developmental stage has been first developed in rodent malaria parasites, such as *P. berghei*^17^*,* followed by *P. knowlesi*^18^ and *P. falciparum*^2,15,19^. This method using full mature schizonts likely allows for delivering foreign DNA into *P. falciparum* more efficiently than other methods using the ring form^6^ or the DNA-preloaded RBCs^7^. However, it was unclear whether its efficiency was sufficient for our proposed CRISPR/Cas9 system using the Cas9-expressing parasite and a linear donor template. To examine this, we quantitatively evaluated the efficiency of the transfection method using full mature schizonts. Since, RBCs containing fully mature schizonts of *P. falciparum* rupture spontaneously in vitro, there are only a few numbers of full mature schizonts in culture: fully mature schizonts usually account for less than 0.05% of total parasites. Thus, we enriched fully mature schizonts by tightly synchronizing the cell cycle of parasites. Briefly, mature and immature schizonts were purified using a Percoll-sorbitol gradient and subsequently cultured for 4 h with fresh RBCs, followed by treatment with 5% sorbitol. The cell cycle of the parasites was synchronized to a window of 4 h with these procedures. We further repeated those procedures two times and eventually obtained tightly synchronized parasites. The final ratio of fully mature schizonts to total parasites increased to approximately 1–2%, demonstrating 20- to 40-fold enrichment (Fig. 1A). Subsequently, the fully mature schizonts were purified along with immature schizonts and then used for transfection. The purified parasites (1.0 × 10^8^ cells) containing the fully mature schizonts were electroporated with 5 μg of the centromere plasmid pFCENv1^20^ by the Nucleofector II system; transgenic parasites were detected 10 days post-transfection, and the number of independently transfected parasites was calculated to be 3.6 × 10^3^ based on the multiplication rate (3.7/cell cycle) and percentage of parasitemia at 12 days post-transfection (Fig. 1B). In contrast, when we transfected parasites with similar amounts of pFCENv1 using DNA-preloaded RBCs, the number of independently transfected parasites was only 29 (Fig. 1B). These results indicated that the method using fully mature schizonts increased the efficiency of DNA introduction more than 125-fold compared to that using DNA-preloaded RBCs. When 50 μg of pFCENv1 was used, the number of transfected parasites reached 2.6 × 10^4^ (Fig. 1B). When 1.0 × 10^3^–1.0 × 10^4^ of rodent malaria parasite (*P. berghei*) were transfected independently, it was sufficient for genetic modification by the CRISPR/Cas9 system using the Cas9-expressing parasite and the liner donor template^13,17^*.* Based on these quantitative comparison, we considered that the method using fully mature schizonts could be used for the improvement of CRISPR/Cas9 system in *P. falciparum*.

### Generation of *P. falciparum* that constitutively expressed Cas9

**Figure 2:**
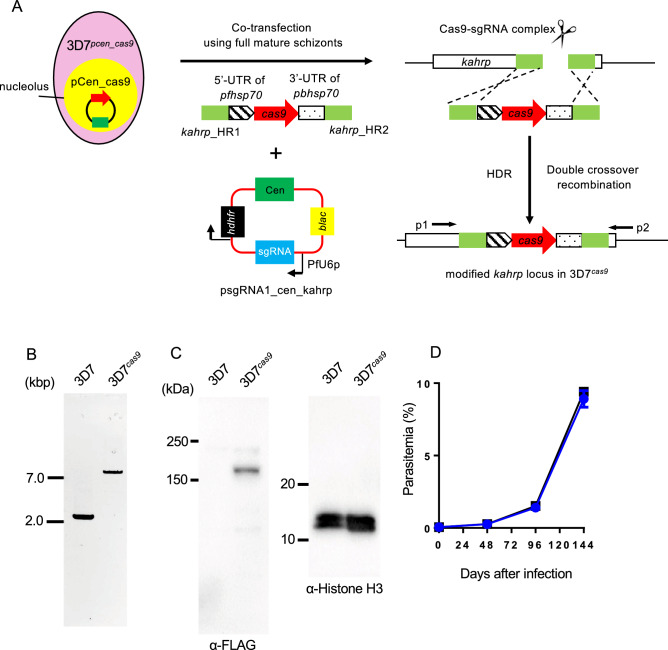
Generation of 3D7^*cas9*^. (**A**) The 3D7^*pcen_cas9*^ parasite that maintained the pCen_cas9 plasmid was co-transfected with the Cas9 expression cassette and the psgRNA_kahrp_cen plasmid using their fully mature schizonts. The expression cassette was integrated into the *kahrp* locus. (**B**) Genotyping PCR of 3D7^*cas9*^ parasites was performed using the p1 and p2 primers. Full length gel image is included in Fig. S4A. (**C**) Expression of Cas9 nuclease was confirmed by Western blot analysis using the anti-FLAG antibody. Histone H3 was used as an internal control. Full length blots are included in Fig. S4B. (**D**) The growth of 3D7^*cas9*^ parasites (blue, circle) during asexual development in RBCs was comparable to that of wild-type parasites (black, box). Positive and negative errors were calculated from the standard error of the mean from biological triplicates. Distributions for each day were compared using the unpaired *t*-test (not significant).

In the recent report, the *cas9* gene was inserted in the parasite’s genome by the single-crossover recombination to generate the Cas9-expressing parasite^10^. However, this Cas9-expressing parasites will lose the *cas9* gene occasionally by additional intra-chromosomal recombination, which reverts the parasites to the wild-type. To avoid this, we inserted the Cas9-expressing cassette by double-crossover recombination with the centromere plasmid-based CRISPR/Cas9 system developed in our previous study (Fig. 2A)^21^. In this system, the Cas9 was stably pre-expressed in the parasites using the centromere plasmid pCen_cas9, and this Cas9-expressing parasite, 3D7^*pcen_cas9*^, was used as the recipient for transfection. Furthermore, to avoid unexpected recombination, the linear form of the Cas9 expression cassette was used as the donor template. The *Streptococcus pyogenes* Cas9 gene was used for the expression cassette, and its transcription was controlled by the promoter of *pfhsp70* (PF3D7_0818900) and the 3′-UTR of *pbhsp70* (PBANKA_0711900). The nuclear localization signal and the FLAG tag sequences were introduced at the N-terminus of Cas9. The Cas9 expression cassette was flanked with two partial sequences of the *kahrp* gene (PF3D7_0202000), which was used as the target genomic locus. The KARHP is one of components of knob structure on the surface of infected RBCs and is not essential for the asexual development of parasite in RBCs in vitro. Thus, the integration of the Cas9 expression cassette at the *kahrp* locus would not affect on the growth of the resultant transgenic parasite in RBCs in vitro. The guide RNA (gRNA), specific for the *kahrp* gene, was cloned into the psgRNA1_cen plasmid, which contains the centromere of *P. falciparum*, and was transcribed by the promoter of U6 spliceosomal RNA (PfU6: PF3D7_1341100) (Fig. 2A). The resultant plasmid was named psgRNA1_cen_kahrp. Twenty-five micrograms of each linearized Cas9 expression cassette and the psgRNA1_cen_kahrp plasmid were co-introduced into fully mature 3D7^*pcen_cas9*^ schizonts by electroporation. Transfection experiments were carried out in duplicate to obtain biologically independent transgenic parasites. Since the psgRNA1_cen_kahrp and pCen_cas9 plasmids had human dihydrofolate reductase and blasticidin deaminase genes, respectively, as drug-selectable markers, transgenic parasites that had two plasmids were screened by treatment with those two drugs. The transgenic parasites became visible in the culture approximately 4 weeks after treatment and were then harvested. To examine whether the Cas9 expression cassette was incorporated in the *kahrp* locus, we analysed their genotypes by PCR using the primer set p1 and p2 (Supplementary Data 1). The results showed specific amplification of a 7.2-kb DNA fragment, indicating the incorporation of the Cas9 expression cassette (Fig. 2B). Subsequently, to remove the psgRNA1_cen_kahrp and pCen_cas9 plasmids from the obtained transgenic parasites, we cultured them in the absence of drug for 6 weeks. Following long-term cultivation, we cloned plasmid-free transgenic parasites, *i.e.,* drug-selectable marker-free parasites, by the limiting dilution procedure. We eventually obtained 4 parasite clones that lost both the pCen_cas9 and psgRNA1_cen_kahrp plasmids. We selected one of these plasmid-free clonal parasites and named it 3D7^*cas9*^. To confirm whether the Cas9 expression cassette was integrated only at the *kahrp* locus in 3D7^*cas9*^, we performed Southern hybridization analysis using the Cas9 gene as probe DNA. The signal was detected solely at 4.8 kb in 3D7^*cas9*^, indicating that the Cas9 expression cassette was integrated as a single copy at the *kahrp* locus in the genome (Fig. S1). Western blot analysis using a FLAG antibody confirmed that the Cas9 was expressed without any degradation (Fig. 2C). The 3D7^*cas9*^ parasites could multiply in RBCs at growth rates comparable to that of the strain 3D7 (Fig. 2D): the multiplication rates of 3D7^*cas9*^ and strain 3D7 were estimated to be 5.2 and 5.3 per cell cycle, respectively. Female and male gametocytes of 3D7^*cas9*^ were detected microscopically; in addition, exflagellation of the male gamete was induced by xanthic acid (Supplementary Mov. 1). These results showed that there was no obvious defect in asexual or sexual development in 3D7^*cas9*^ due to the constitutive expression of Cas9.

To examine the effect of the constitutive expression of Cas9 on genome integrity, we conducted whole-genome sequencing analysis of the 3D7^*cas9*^ parasite and examined the accumulation of mutations caused by Cas9 during maintenance. The genomic DNA used for analysis was purified from 3D7^*cas9*^ that had been maintained over one month in culture and then sequenced to a depth of approximately 62.9 × coverage, followed by comparison to the reference genome sequence of *P. falciparum* strain 3D7 deposited in PlasmoDB (https://plasmodb.org/plasmo/). A total of 165 SNPs and indels were called (Supplementary data 2), and 127 of them were found in intergenic regions, subtelomeric regions, and introns (Supplementary data 3). The SNPs and indels called in those regions may have been false positives because mapping errors frequently occur in these regions due to their low sequence complexity. Although 38 clear SNPs and indels were called in 3D7^*cas9*^, they might not have been caused by the constitutive expression of Cas9. The 3D7^*pcen_cas9*^ used for the generation of 3D7^*cas9*^ in this study had been cultured for a long time, e.g., more than two months, which allowed for the accumulation of mutations that did not participate in multiplication in RBCs. As shown later, these mutations are commonly found in the transgenic parasite, supporting our speculation (Supplementary data 2). We further conducted copy number variation analysis of 3D7^*cas9*^ and identified increase of copy numbers in four genomic regions (Supplementary data 4). There were 10 genes in those identified regions and all of them were not involved in asexual development in RBCs. These increase of copy numbers were commonly identified in the transgenic parasites as in the case of SNPs (Supplementary data 4). Therefore, we concluded based on those results that the constitutive expression of Cas9 did not cause unexpected mutations in the parasite genome.

### Genetic modification using 3D7^*cas9*^ and a linear donor template

**Figure 3:**
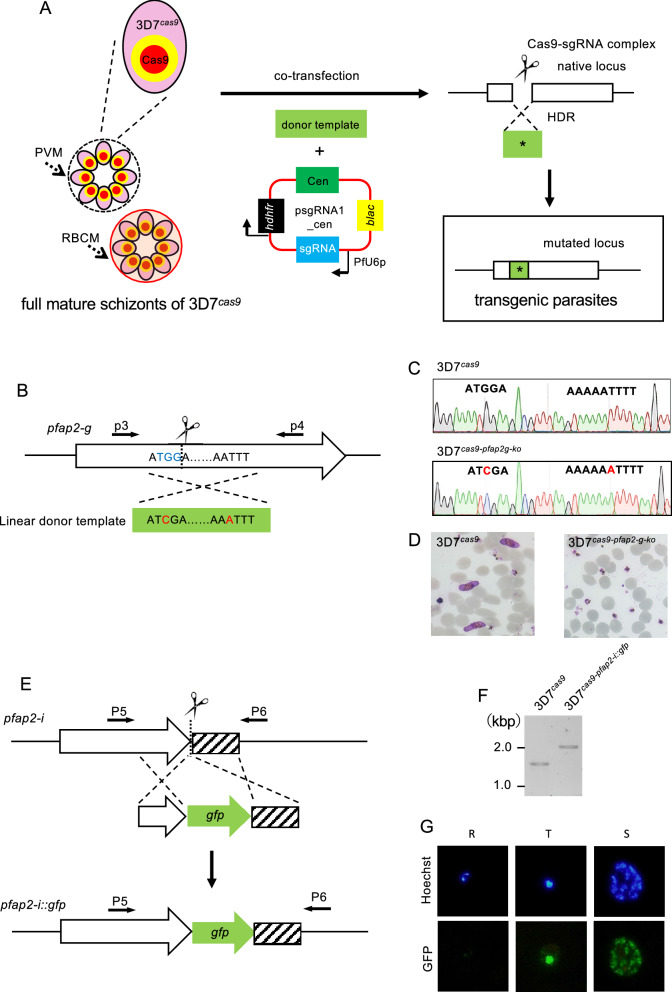
Genetic engineering using 3D7^*cas9*^. (**A**) To engineer the gene, 3D7^*cas9*^ was co-transfected with the linear form of donor template DNA and psgRNA1_cen containing the sgRNA. The targeted genomic locus was cleaved by the Cas9-sgRNA complex, followed by HDR with the donor template. (**B**) *pfap2-g* was disrupted by insertion of a single adenosine in the open reading frame. The PAM sequence TGG was also mutated by substitution from guanosine to cytosine. (**C**) The mutations introduced in *pfap2-g* were confirmed by sequencing. Red indicates the mutation. (**D**) The 3D7^*cas9-pfap2-g-ko*^ parasite completely lacks gametocyte production. (**E**) The *gfp* gene was integrated at the C-terminus of PfAP2-I. (**F**) Genotyping PCR of 3D7^*cas9-pfap2-i::gfp*^ parasites was performed using the p5 and p6 primers to examine the integration of *gfp*. Full length gel image is included Fig. S4D. (**G**) PfAP2-I-GFP expression in 3D7^*cas9-pfap2-i::gfp*^ was uniquely confirmed in trophozoite (T) and schizont (S), not in ring form (R).

Next, we attempted to engineer a gene by co-introducing the linear donor template and the plasmid carrying the sgRNA into the fully mature schizonts of 3D7^*cas9*^ (Fig. 3A). As an initial attempt, we introduced a single nucleotide insertion in the coding sequence of the transcription factor PfAP2-G, which is involved in gametocytogenesis (Fig. 3B). The sgRNA designed in the middle of its AP2 domain was cloned in the psgRNA1_cen plasmid, and the resultant plasmid was named psgRNA1_cen_ap2g. The linear donor template with single nucleotide insertion was generated by PCR. In addition, to prevent re-cleavage by the Cas9-sgRNA complex after homologous recombination, a shield mutation was introduced into the PAM sequence in the donor template DNA. The fully mature schizonts of 3D7^*cas9*^ were purified using a Percoll-sorbitol gradient and then co-transfected with 25 μg each of psgRNA1_cen_ap2g and the linear donor template DNA. The transfected parasites were maintained after electroporation in the absence of drug for 3 days, followed by pyrimethamine treatment for 10 days. The transgenic parasites were visible in the culture 2 days after withdrawal of drug and then harvested. The target region was amplified from genomic DNA purified from harvested parasites using primers p3 and p4 and sequenced (Fig. 3B). This analysis confirmed that shield mutations were introduced with almost 100% efficiency: the wild-type PAM sequence was not detected in this analysis (Fig. S2). However, some of the harvested parasites did not have an additional A residue between nucleotide positions 6563–6564: we detected minor chromatograms of the wild-type sequence downstream of nucleotide position 6563 (Fig. S2). These results suggested that cleavage and repair of the target site occurred in almost all the obtained transgenic parasites, and the majority of them had both shield mutations and inserted A residues, although there was a minor population that possessed only shield mutations. In these parasites harbouring only shield mutations, HDR with a linear donor template might have completed before reaching the site where a single nucleotide was inserted. Seven clonal parasites were established by the limiting dilution procedure, and their mutations were then examined by sequencing analysis (Fig. 3C). This analysis showed that all of them possessed the shield mutation, but one clonal parasite did not have an A nucleotide residue, supporting the possibility described above. We estimated the efficiency of this genetic manipulation to be 85% based on this result. The clonal parasites with disruption of *pfap2-g*, named 3D7^*cas9-pfap2-g-ko*^*,* completely lacked gametocyte production capability (Fig. 3D).

Subsequently, we examined by whole-genome sequencing whether any off-target sites were mutated in 3D7^*cas9-pfap2-g-ko*^. A total of 170 SNPs and indels were called except for the single nucleotide insertion and the shield mutation in the *pfap2-g* gene by comparison to the genomic sequence of the parental 3D7^*cas9*^ parasite (Supplementary Data 2 and 5). In total, 165 SNPs and indels were shared between 3D7^*cas9-pfap2-g-ko*^ and 3D7^*cas9*^, indicating that they were inherited from the parental 3D7^*cas9*^. This analysis further called two indels unique in the exons of PF3D7_0505000 and PF3D7_0818700. Both indels were found in repetitive sequences; in addition, no sequences around the indels were similar to the sgRNA, which suggested that they were false positives due to low sequence complexity. Therefore, we concluded that no off-target mutations were caused by genetic engineering using our CRISPR/Cas9 system.

In addition to single nucleotide insertion, we performed another type of genetic engineering: fluorescent protein tagging (Fig. 3E). We fused GFP with the transcription factor PfAP2-I, which is essential for asexual multiplication. The sgRNA was designed at the region proximal to its terminal codon and cloned in the psgRNA1_cen plasmid, resulting in the psgRNA1_cen_ap2-i plasmid. The linear donor template encoding the *gfp* gene and the psgRNA1_cen_ap2-i plasmid were co-introduced into 3D7^*cas9*^. The transgenic parasites emerged 2 days after drug treatment for 10 days. To examine the fusion of *pfap2-i* with *gfp*, PCR analysis of the harvested parasites was performed using the primer sets P5 and P6. The results showed the amplification of an approximately 2.0-kbp fragment derived from the modified genomic locus, which confirmed GFP fusion (Fig. 3F). In contrast, the estimated 1.0 kbp fragment from wild-type parasites was not amplified in the pooled parasite population, suggesting that GFP was fused to PfAP2-I with almost 100% efficiency. We subsequently cloned parasites by the limiting dilution procedure and then named them 3D7^*cas9-pfap2-i::gfp*^. Sequencing analysis around the C-terminus of PfAP2-I in the 3D7^*cas9-pfap2-i::gfp*^ parasite showed the correct integration of the coding sequence of GFP in frame (Fig. S3A,B). Southern hybridization analysis of the clonal parasite detected a single signal at 4.0 kbp, which was consistent with the expected restriction map of the 3D7^*cas9-pfap2-i::gfp*^ parasite (Fig. S3C). Furthermore, Southern analysis using the *gfp* gene as the probe DNA detected a signal at 5.0 kbp, indicating that *gfp* was integrated only at the C-terminus of PfAP2-I (Fig. S3D). The 3D7^*cas9-pfap2-i::gfp*^ parasite expressed GFP in the nuclei of trophozoites and schizonts, which confirmed its proper localization and expression profile (Fig. 3G). Collectively, these genes could be modified by co-transfecting 3D7^*cas9*^ with linear donor template DNA and plasmids containing sgRNA without unexpected recombination, showing that the technical problems of the current CRISPR/Cas9 system in *P. falciparum* could be solved.

### Double genetic engineering using the improved CRISPR/Cas9 system

**Figure 4:**
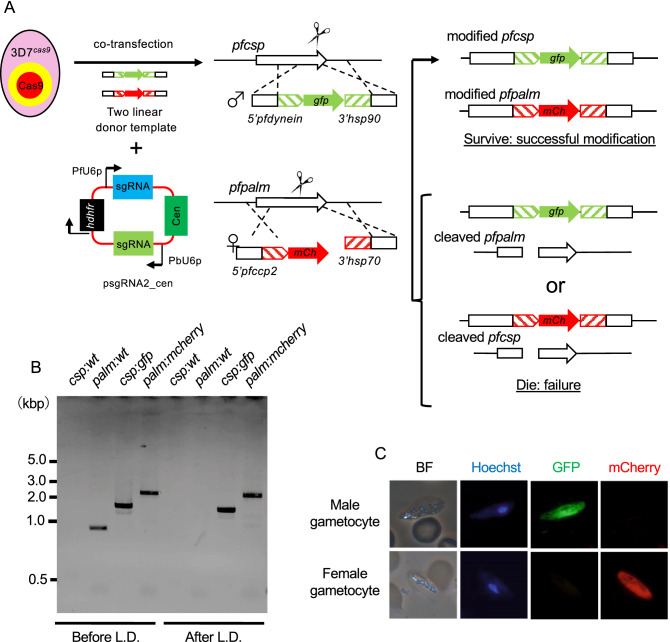
Generation of a reporter parasite line of sexual parasites by double genetic engineering. (**A**) Two linear donor templates, which contained male- and female-specific reporter cassettes, and the psgRNA2_cen plasmid containing two sgRNAs were co-introduced into 3D7^*cas9*^ parasites. A male-specific reporter cassette with the *gfp* gene was integrated at the *pfcsp* locus. A female-specific cassette with the *mCherry* gene was performed at the *pfpalm* locus. If the cleaved genomic loci at *pfcsp* and *pfpalm* were repaired with donor templates by HDR, the parasite would survive. However, if one of them was not repaired, parasites would die due to instability of the cleaved chromosome. (**B**) Genotyping PCR was performed using genomic DNA purified from 3D7^*cas9-Pfg_red/green*^ before and after limiting dilution. The primers used for this analysis are shown in Supplementary data 1. Full length gel image is included in Fig. S4G. (**C**) GFP and mCherry were expressed in male and female gametocytes of 3D7^*cas9-Pfg_red/green*^, respectively.

Our sequence analysis showed that the wild-type parasites were not present in the parasite population emerging in culture after co-transfection with the linear donor template and plasmid DNA containing the sgRNA. *P. falciparum* does not have the canonical nonhomologous end joining (cNHEJ) pathway^22^; if a double-strand break is not repaired by HDR using a donor template, the parasite will die, probably due to instability of the cleaved chromosome, resulting in the observed elimination of wild-type parasites. Hence, if multiple genomic sites are cleaved by Cas9 with sgRNA corresponding to each target site, only transgenic parasites in which all sites are repaired by HDR may survive, resulting in multiple genetic modifications. To validate this concept, we modified two genes simultaneously by transfecting 3D7^*cas9*^ with two sgRNAs and two linear donor templates (Fig. 4A). To this end, we generated the centromere plasmid psgRNA2_cen, which expressed two sgRNAs. Each sgRNA including tracrRNA was transcribed by the promoters of U6 spliceosomal RNA of *P. falciparum* and *P. berghei* (PbU6: PBANKA_1354380). In this attempt, we introduced expression cassettes of two fluorescent proteins, GFP and mCherry, into two genomic loci on different chromosomes; the GFP and mCherry expression cassettes were integrated into the *pfcsp* gene on chromosome 3 and the *pfpalm* gene on chromosome 6, respectively. Moreover, the *gfp* and *mcherry* genes were transcribed sex-specifically under the control of the promoters of the dynein heavy chain (Male: PF3D7_1023100) and CCP2 (Female: PF3D7_1455800), respectively. The expression cassettes of GFP and mCherry were flanked with sequences used for HDR by PCR, resulting in each donor template DNA. The gRNAs specific for the *pfcsp* and *pfpalm* genes were designed and cloned into the psgRNA2_cen plasmid, resulting in psgRNA2_cen_csp:palm. The psgRNA2_cen_csp:palm plasmid and the two donor templates containing GFP and mCherry expression cassettes were co-introduced into the 3D7^*cas9*^ parasites. Transgenic parasites were harvested after becoming visible in the culture 14 days after transfection. PCR-based genotype analysis indicated that the GFP cassette was integrated into the genomic locus of the *pfcsp* gene with almost 100% efficiency but the mCherry cassette with lower efficiency; the fragments were amplified from not only the modified *pfpalm* locus (2.8 kbp) but also the wild-type *pfpalm* locus (1.1 kbp) (Fig. 4B). We considered that this less efficient mCherry fusion was probably due to less efficient cleavage of the Cas9-sgRNA complex for the *pfpalm* locus. Subsequently, we cloned the transgenic parasite 3D7^*cas9-Pfg_red/green*^, in which both GFP and mCherry protein expression cassettes were integrated into the corresponding locus, by limiting dilution. The integration of both cassettes was confirmed in the 3D7^*cas9-Pfg_red/green*^ parasites by PCR and sequence analyses. In addition, fluorescence microscopic analysis showed that male and female gametocytes of 3D7^*cas9-Pfg_red/green*^ expressed GFP and mCherry proteins, respectively (Fig. 4C); in contrast, there was no fluorescence in parasites at asexual stages, such as the ring form, trophozoite, and schizont stages. These results demonstrated that multiple genetic modifications could be carried out simultaneously by utilizing the CRISPR/Cas9 system developed in this study.

## Discussion

In the present study, we improved the CRISPR/Cas9 system by using the Cas9-expressing parasite and the linear donor template. Although some individual methods either using the Cas9-expressing parasite or the linear donor template have been developed, the technical issues still remained as described in the introduction. Our method allows for generating the desired transgenic parasites within approximately 2 weeks with high efficiency (> 85%), and no unexpected recombination was observed in the resultant parasites. We consider based on these results that the usage of the Cas9-expressing parasite and the linear donor template in combination is important for improving the efficiency and the robustness of CRISPR/Cas9 system in *P. falciparum*.

The linear form of DNA has to be used to avoid unexpected recombination; however, it is readily lost from the parasites during nuclear division due to its low segregation, probably disappearing from the parasite during the first cell cycle after electroporation. Thus, for genetic engineering using a linear donor template, the HDR between the cleaved genome and the linear donor template must be completed as quickly as possible after transfection. To this end, a linear donor template has to be transferred with high efficiency, but transfection methods using the ring form of the parasite or the DNA-preloaded RBCs are not efficient enough. Therefore, we consider that the transfection method^2,15,19^ using fully mature schizonts is essential for the CRISPR/Cas9 system using a linear donor template. In addition, we consider that the pre-expression of Cas9 in the parasite could assist for the genetic engineering by the linear donor template. The pre-expressed Cas9 can form a complex with sgRNA immediately after the introduction of the plasmid carrying sgRNA, and this immediate cleavage prompts the subsequent HDR with the linear donor template by efficiently recruiting the molecule responsible for recombination. Thus, pre-expression of Cas9 induce efficient recombination possibly, allowing the completion of HDR before the parasite loses the template DNA.

High efficiency of DNA transfer into the parasite can be achieved by using fully mature schizonts^2,15,19^. In contrast to fully mature schizonts, immature schizonts are sensitive to electroporation and thus readily die from electric pulses, resulting in the failure of DNA introduction. The fully mature schizonts contain invasive merozoites, which are released as a result of the disruption of two membranes, belonging to parasitophorous vacuoles (PVMs) and RBCs (RBCMs). The merozoites are wrapped with either PVM or RBCM, suggesting that both become fragile during schizont maturation, including proteolytic digestion of membrane proteins^23^, and that one membrane is retained by chance. This remaining membrane may be readily disrupted by electroporation, and transfected merozoites may invade new RBCs immediately, resulting in high transfection efficiency.

The Cas9 nuclease-sgRNA complex binds to double-stranded DNA even if there are three to five base pair mismatches in the PAM-distal region of the sgRNA sequence. Thus, it can cleave other genomic sequences, *i.e.,* off-target sites, other than the desired target site. This cleavage at off-target sites is repaired in eukaryotic cells by the cNHEJ pathway, causing a small deletion or insertion. On the other hand, the *Plasmodium* genus, including *P. falciparum,* lacks the cNHEJ pathway. Thus, if off-target sites are cleaved in *Plasmodium* parasites, they will not be repaired by the cNHEJ pathway. These off-target cleavages make the genome unstable, resulting in the death of parasites. As a result, parasites with off-target cleavages may be eliminated from the transgenic parasite population. The whole-genome sequencing in this study suggested that there were no off-target mutations in the resultant transgenic parasite clone. Furthermore, similar results were obtained in our previous study in the rodent malaria parasite *P. berghei*^13^. Therefore, we consider that genetic engineering can be performed by the CRISPR/Cas9 system without off-target mutations in the *Plasmodium* genus.

Genetic modification at two different genomic loci was achieved performed by our method. In the present study, we used this method to integrate two fluorescence protein expression cassettes into different chromosomes. In addition, it can be used for various genetic modifications, such as double gene targeting and tagging and gene targeting of two different genes. Furthermore, this method will be useful for deleting or replacing kbp-scale genomic regions, which has been difficult to accomplish by using one sgRNA. In general, after the target sites are cleaved by Cas9, the DNA sequence around the 5′ end on either strand is trimmed, generating 3′ overhangs. These overhangs invade the complementary donor template, initiating HDR. When one sgRNA is used for kbp-scale genetic modification, there is a distance between the cleaved genomic locus and the regions used for HDR. Due to this distance, it is difficult to generate overhangs possessing complementary sequences to the regions used for HDR, resulting in failure. However, when two sgRNAs are used for similar genetic modification, each cleaved genomic locus will be proximal to the regions used for HDR. The 3′ overhang sequences that are complementary to the region used for HDR will be readily generated in this case, resulting in successful modification. Kilobase-scale genetic modifications can be utilized for a wide range of experiments, such as generating complete null mutants by deletion of entire gene regions, including the coding region, 5′-, and 3′-UTR; replacing promoter regions with a synthetic DNA fragments; and deleting specific genomic loci with unique epigenetic marks. Thus, we anticipate that genetic modification using two sgRNAs will be useful for generating transgenic parasites with complex genetic modifications.

When GFP and mCherry expression cassettes were integrated into the *pfcsp* and *pfpalm* loci, respectively, by our method using the psgRNA2_cen plasmid, we found that some transgenic parasites maintained the wild-type *pfpalm* sequence, including the site targeted by the sgRNA. This suggested that cleavage by the Cas9-sgRNA complex was less efficient at the *pfpalm* gene than at the *pfcsp* gene. Because the sgRNA for *pfpalm* was controlled under the PbU6 promoter, its transcriptional activity in *P. falciparum* might be weaker than that of the PfU6 promoter. This weaker transcriptional activity of the PbU6 promoter might cause less efficient cleavage of the targeted sequence of the *pfpalm* gene, resulting in failure of integration of mCherry cassette. We consider that the transcriptional activity of the promoter used for the sgRNA may be a determinant of the efficiency of genetic engineering by the CRISPR/Cas9 system. Thus, an appropriate promoter derived from *P. falciparum* should be used for the transcription of sgRNA.

In conclusion, our method based on the CRISPR/Cas9 system overcame technical issues in the current methods for *P. falciparum*. Furthermore, since our approach dramatically elevates the efficiency with which transgenic parasites were generated, it does not only allow simple genetic modification, such as site-directed mutagenesis, but may also enable us to perform complicated gene editing, such as editing two loci at once or achieving large-scale editing, which has never been accomplished with previous methods. If a similar method could be developed in strain NF54, which is widely used for parasite transmission experiments in mosquito vectors, the functional analysis of genes would be expanded throughout the life cycle. In such attempt, the other locus, e.g. p47 (PF3D7_1346800), might be better than the *kahrp* locus used in this study, because the disruption of *kahrp* gene will cause the loss of function of the knob associated proteins, such as PfEMP1. We expect that our method will open new avenues in molecular genetics and post-genomics of *P. falciparum* and will become the standard method for genetic modification of the parasite.

## Materials and methods

### Parasites and culture

The 3D7^*pcen_cas9*^ parasite, which contains the *cas9* expression centromere plasmid pfCas9_cen, was generated from *P. falciparum* strain 3D7 in our previous study^21^ and used for the generation of the 3D7^*cas9*^ parasite in the present study. The 3D7^*cas9*^ parasite will be deposited at the Malaria Research and Reference Reagent Resource Center, MR4 (https://www.beiresources.org/About/MR4.aspx). All parasites were cultured in vitro under low oxygen concentrations as described previously^20^.

### Transfection of fully mature schizonts

Parasites were roughly synchronized by treatment with 5% sorbitol prior to tight synchronization. When most of the parasites had developed into schizonts, they were purified using a 40–70% discontinuous Percoll gradient solution (GE Healthcare Life Sciences) with 6% sorbitol. Purified schizonts were cultured with fresh RBCs for four hours and then treated with 5% sorbitol. The resulting parasites were synchronized within a window of approximately four hours. These Percoll and sorbitol synchronizations were repeated three times, resulting in tightly synchronized parasites. The emergence of fully mature schizonts was monitored via microscope for 88 h after the final synchronization, and the ratios of mature schizonts to total schizonts were determined every two hours. When the ratio of fully mature schizonts to total schizonts reached a maximum number, the parasites were purified again using a discontinuous Percoll gradient. Purified schizonts consisted of both immature and fully mature forms, and the ratio of fully mature schizonts usually reached approximately 1–2%. The DNA samples, e.g., 25 μg of each linear donor template and the plasmid containing the sgRNA, were dissolved in 100 μl of Parasite Nucleofector II solution (LONZA) and mixed with the purified schizonts (1.0 × 10^8^). The parasites were electroporated using the U-033 program on a Nucleofector II device (LONZA). Immediately after electroporation, transfected parasites were mixed with 0.1 ml of complete medium, which consisted of RPMI-1640 containing 10% human serum (obtained from the Japanese Red Cross Osaka Blood Center), 10% AlbuMAX II (GIBCO BRL), 25 mM HEPES, 0.225% sodium bicarbonate, and 0.38 mM hypoxanthine supplemented with 10 μg/ml gentamicin, and then cultured in 5 ml of complete medium with fresh RBCs. Drug selection of transgenic parasites was initiated 72 h after transfection and continued for 10 days. Recombination was confirmed by PCR and Sanger sequencing, and then clonal parasites were obtained by limiting dilution. To evaluate the transfection efficiency, the number of independently transfected parasites was estimated based on the percentage of parasitemia and the multiplication rate of the transgenic parasite. The multiplication rate (3.7 per cell cycle) of the transgenic parasite with the introduced pFCENv1 was determined in the presence of pyrimethamine, as in our previous study^20^. The number of independently transfected parasites was calculated using the following equation:

1 $${\text{T}} \times {\text{P}}/100 = \left[ {{\text{I}} \times \left( {3.7} \right)^{{{\text{D}}/2}} } \right]$$

where T is the total number of RBCs in culture (5 ml medium with Ht 2%); D is the number of days after transfection; P is the percentage of parasitemia at day D; and I is the number of independently transfected parasites.

### Construction of sgRNA-expressing plasmid

The gRNA was designed as described previously^13^. Briefly, a 19-bp gRNA target sequence, which is predicted to have no off-target candidates, was designed using CHOPCHOP program (https://chopchop.cbu.uib.no/). A guanosine was added at 5′ end of the designed sequence since the U6 promoter requires a guanosine nucleotide to initiate transcription. To insert the 20-bp target sequence into plasmids, two single-stranded oligonucleotides were synthesized and annealed with each other to generate double-stranded oligonucleotides with overhangs used for cloning into *Bsm*BI- or *Bsa*I-digested plasmids as described below.

A centromere plasmid for expressing sgRNA was generated from the pf-gRNA plasmid^21^. The pf-gRNA contains a sgRNA expression cassette in which transcription of sgRNA is controlled by the PfU6 (U6 spliceosomal RNA, PF3D7_1341100) promoter. Two recognition sites of *Bsm*BI are introduced between the PfU6 promoter and tracrRNA and used for cloning the gRNA. This plasmid also contains *hdhfr*, a drug-selectable marker gene, which is driven by the *P. berghei elongation factor 1α* (PBANKA_1133300 and PBANKA_1133400) promoter. The centromere of *P. falciparum* chromosome 5 was excised from the pCen_cas9 plasmid^21^ by *Bam*HI and *Not*I digestion and then cloned into pf-gRNA, resulting in the psgRNA1_cen plasmid. The annealed gRNA oligonucleotides were cloned into the *Bsm*BI-digested psgRNA1_cen plasmid.

To generate a centromere plasmid expressing two sgRNAs targeting different genomic loci, another sgRNA expression cassette was incorporated into the psgRNA1_cen plasmid. The sgRNA expression cassette was amplified from the psgRNA2 plasmid previously reported by Shinzawa et al.^13^. The cassette is composed of the PbU6 (U6 spliceosomal RNA, PBANKA_1354380) promoter and tracrRNA scaffold, and two *Bsa*I recognition sites are included between them to clone the gRNA. The β-lactamase gene, which is a well-known selectable marker in *E. coli*, contains the *Bsa*I site, which was eliminated by introducing a synonymous mutation before cloning the sgRNA expression cassette. The PbU6-driven sgRNA cassette was then integrated into the mutated psgRNA1-cen at the *Bam*HI site by In-Fusion cloning, and the resulting plasmid was named psgRNA2_cen. Two gRNAs were cloned into *Bsm*BI and *Bsa*I, and the resultant plasmid expressing two sgRNAs was used for the multiple genetic modification experiments. The sequences of all oligonucleotides used in this study are provided in Supplementary Data 1. Both of psgRNA1_cen and psgRNA2_cen plasmids will deposited at MR4.

### Preparation of donor template DNA

The *pfhsp70* (PF3D7_0818900) promoter and the *cas9* gene with the 3′-UTR of *pbhsp70* (PBANKA_0711900) were amplified from the genomic DNA of strain 3D7 and the pfCas9_cen plasmid, respectively. These two DNA fragments were then fused by overlap PCR, digested with *Bam*HI and *Sal*I, and cloned in tandem into *Bam*HI and *Sal*I-digested pBluescript SK(+) using a DNA Ligation Kit (Takara). Two partial sequences were amplified from the *kahrp* locus and cloned into the plasmid containing the *cas9* expression cassette. These sequences flanked the *cas9* expression cassette on both sides. The resultant plasmid with the Cas9 expression cassette and two partial sequences of *kahrp* was linearized by digestion with *Kpn*I and *Not*I restriction enzymes and used as donor template DNA to generate the 3D7^*cas9*^ parasite.

A donor DNA template for *pfap2-g* (PF3D7_1222600) gene knockout was produced by overlap PCR. The donor template DNA contained the following two mutations: an adenosine insertion at position 6563 of *pfap2-g* and a single nucleotide substitution at the PAM sequence. For fusion of *gfp* to *pfap2-i* (PF3D7_1007700), donor template DNA containing the *gfp* gene flanking two homologous regions of *pfap2-i* was produced by overlap PCR. Six nucleotides encoding Ala and Ser residues were introduced between *pfap2-i* and *gfp* as a linker sequence. The male- and female-specific reporter cassettes were generated using GFP and mCherry, respectively. The *pfdynein* (dynein heavy chain, PF3D7_1023100) and *pfccp2* (LCCL domain-containing protein, PF3D7_1455800) promoters were used as male- and female-specific promoters, respectively. The *pfcsp* (circumsporozoite protein, PF3D7_0304600) locus was used as the target site for integration of the male-specific reporter cassette with GFP. The *pfpalm* (liver merozoite formation protein, PF3D7_0602300) locus was used as the target site for the female-specific reporter cassette with mCherry. The transcription of *gfp* and mCherry was terminated by the 3′-UTRs of *pfhsp90* (PF3D7_0708400) and *Pfhsp70*, respectively. DNA fragments of the male- and female-specific reporter cassettes were generated by overlap PCR. The male-specific reporter cassette was then cloned into the *Eco*RV recognition sites in pBluescript SK(+). Two partial sequences of *pfcsp* were amplified from genomic DNA of strain 3D7 and cloned on each side of the male-specific reporter cassette in the plasmid using In-Fusion cloning kit. The female-specific reporter cassette was cloned into pBluescript SK(+) digested with *Xho*I and *Hind*III, and two partial sequences of *pfpalm* were also amplified and cloned at each side of the female-specific reporter cassette in a similar manner as the male-specific cassette. The male- and female-specific reporter cassettes flanking those sequences used for HDR were amplified from the resultant plasmids by PCR, and the resultant linear DNA fragments were used for the transfection experiment.

### Southern blot analysis

Genomic DNA used was purified from 3D7^*cas9*^ parasites by the standard phenol/chloroform method^20^. Briefly, parasite pellets were dissolved in HMW buffer, which was 10 mM Tris–HCl, pH 8.0, 150 mM NaCl, 10 mM EDTA, and 0.1% SDS, and then treated with 40 μg/ml RNase (Takara) for 30 min, followed by treatment with 200 μg/ml Proteinase K (Wako) for 90 min. Genomic DNA was extracted once with phenol, followed by extraction with phenol–chloroform-isoamyl alcohol. After precipitation with ethanol, the DNA was dissolved in TE buffer. Genomic DNA was digested with *Eco*RI and *Eco*RV for 8 h. The digested DNA was separated on 1% agarose gels and blotted onto nitrocellulose membranes (Amersham Hybond-N + , Merck). Probe DNA labelling and detection were carried out using DIG High Prime DNA Labeling and Detection Starter Kit II according to the manufacturer’s instructions. The hybridized signals were detected using ChemiDoc MP (Bio-Rad). All other Southern hybridization analyses were performed in a similar manner as described above.

### Western blotting analysis

Infected RBCs were lysed with RBC lysis buffer (150 mM NH_4_Cl, 10 mM NaHCO_3_, and 1 mM EDTA). After RBC lysis, the parasites were recovered by centrifugation and dissolved in 1 × SDS-loading buffer containing 5% 2-mercaptoethanol, followed by boiling for 5 min. Western blotting was performed as described previously. In brief, parasite proteins (1 × 10^7^ parasites per lane) were separated by SDS-PAGE and transferred to a PVDF membrane. The blotted membrane was blocked in TBST containing 4% skimmed milk, incubated for 90 min with primary antibodies in the same buffer, washed and then incubated for 60 min with horseradish peroxidase-conjugated secondary antibody. Mouse anti-FLAG M2 antibody (1:1000; Sigma, F1804-200UG) was used for the detection of the FLAG-tagged Cas9 nuclease. Anti-histone H3 antibody (1:200, Millipore, 055-499) was used as the internal control. HRP-conjugated goat anti-mouse IgG (H + L) (1:10,000, Jackson 115–035-146) was used as a secondary antibody. The HRP signals were visualized using Immobilon Western Chemiluminescent HRP Substrate (Millipore) and detected with ChemiDoc MP (Bio-Rad).

### Evaluation of growth during asexual development

The parasitemia of parasites was adjusted to 0.1% and cultured in complete medium as described previously^20^. The progression of parasitemia was examined every 48 h using Giemsa-stained thin smears. The average parasitemia between 3D7^*cas9*^ and strain 3D7 was evaluated using a t-test. The growth rate was calculated based on the approximate growth curve. The curve was represented by the following equation;

2 $${\text{P}} = {\text{Ae}}^{{{\text{xD}}}}$$

where P is the parasitemia; A is the constant value; D is the day of the post-infection; and e^x^ is the growth rate.

### Whole-genome sequencing and variant calling

Genomic DNAs used for whole-genome sequencing were purified from 3D7^*cas9*^ parasites and 3D7^*cas9-pfap2-g-ko*^ parasites as described above. The obtained genomic DNA was further purified using a NucleoSpin gDNA Clean-up Kit (Macherey–Nagel). Each of the purified genomic DNA samples was sheared to an average size of 600 bp with Covaris S220 (Covaris), and then, from the sheared DNA, DNA libraries were prepared using the KAPA Hyper Prep Kit (KAPA Biosystems) and TruSeq HT adapters (Illumina) according to the manufacturer's instructions. Whole-genome sequencing was performed on the Illumina MiSeq platform (Illumina) with 251-bp and 301-bp single-end sequencing.

Acquired Illumina sequencing reads were filtered using Trimmomatic (version 0.38, http://www.usadellab.org/cms/?page=trimmomatic) to remove low-quality reads. The numbers of obtained reads of 3D7^*cas9*^ parasites and 3D7^*cas9-pfap2-g-ko*^ parasites were 6,021,306 and 12,155,116 after filtration and their qualities obtained by FASTQC analysis are more than 20. The filtered reads were mapped to the *P. falciparum* 3D7 reference (PlasmoDB, version 35) using the BWA-MEM mapping algorithm (version 0.7.17, http://bio-bwa.sourceforge.net) with the default setting. Variant calling was performed using HaplotypeCaller of GATK (version 3.8, https://software.broadinstitute.org/gatk) to detect single nucleotide polymorphisms (SNPs) and insertions and/or deletions (indels). A comparison of variant calls of the parental line, i.e., 3D7^*cas9*^ parasite, and the mutant line, i.e., the 3D7^*cas9-pfap2g-ko*^ parasite, was carried out with GenotypeGVCFs of GATK. Then, SNPs and indels were selected with standard filtering parameters. The variants that were called uniquely in the mutant line were confirmed by mapping using the genome browser IGV (http://software.broadinstitute.org/software/igv/home) to remove false-positive variants. For CNV analysis, we mapped the sequence reads to *P. falciparum* 3D7 reference (PlasmoDB, version 35) using Bowtie2 and removed multiple-mapped reads by grep command. The resultant sam file was converted to bam file by samtools. The number of mapped reads per 1kbp were counted using featureCounts, followed by calculating average of the obtained numbers. The genomic region, where reads were mapped more than 2 folds compared to average, were identified.

### Statistics and reproducibility

For parasite growth during asexual development, the values are presented as the mean ± SEM from at least three biological replicates and were statistically compared using unpaired Student’s t-test. The exact number of biological replicates is provided in individual figure legends. The statistical analyses were performed with GraphPad Prism 6.0 (GraphPad Software Inc.).

## Supplementary Information

Supplementary Video 1.

Supplementary Information 1.

Supplementary Information 2.

Supplementary Information 3.

Supplementary Information 4.

Supplementary Information 5.

Supplementary Figures.

## Data Availability

Whole-genome sequencing data were deposited in the DDBJ database under accession numbers DRA011698. All relevant data are available from the authors upon request.
