# Supplementary material for: Highly efficient CRISPR/Cas9 system in Plasmodium falciparum using Cas9-expressing parasites and a linear donor template
Source: Sci Rep. 2021 Sep 16;11:18501. doi: 10.1038/s41598-021-97984-z (PMC8445982; doi:10.1038/s41598-021-97984-z)
Supplement: Supplementary file 7 — Supplementary Figures. [file 41598_2021_97984_MOESM7_ESM.pdf]

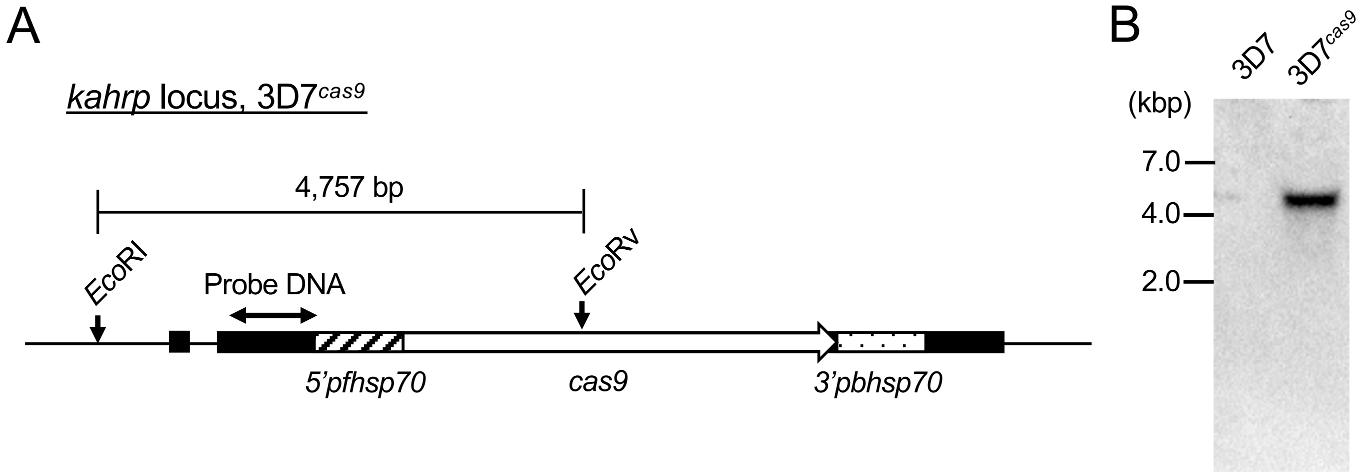

Figure S1. (A) Schematic drawing of *kahrp* locus of 3D7*cas9* parasite. The expression cassette of Cas9 was integrated into the second exon of *kahrp*. Restriction enzyme recognition sites of *Eco*RI and *Eco*RV are indicated by arrows. Those enzymes were used for Southern hybridization analysis in Fig. S1B. In addition, the DNA sequence used for the probe in Southern analysis was shown. (B) Southern hybridization analysis of 3D7*cas9*. The genomic DNA was digested with *Eco*RI and *Eco*RV, followed by hybridizing with the probe DNA, as described in (A). Full length blot is included in Fig. S4C.

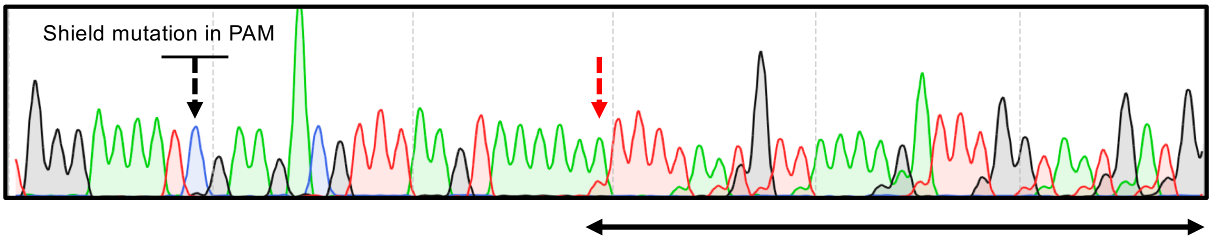

Figure S2. Sequence analysis of 3D7$^{cas9\text{-}pfap2\text{-}g\text{-}ko}$ parasites. The genomic DNA was purified from the parasite harvested before the limiting dilution and then sequenced. The PAM sequence, TGG, was mutated to the TCG almost completely, as indicated by the black arrow. While one adenosine insertion was detected as indicated by the red arrow (refer to Fig. 3C), the minor histograms were also detected. Those minor histograms were frequently detected in downstream of the site where the adenosine was inserted. These were derived from the wild-type sequences.

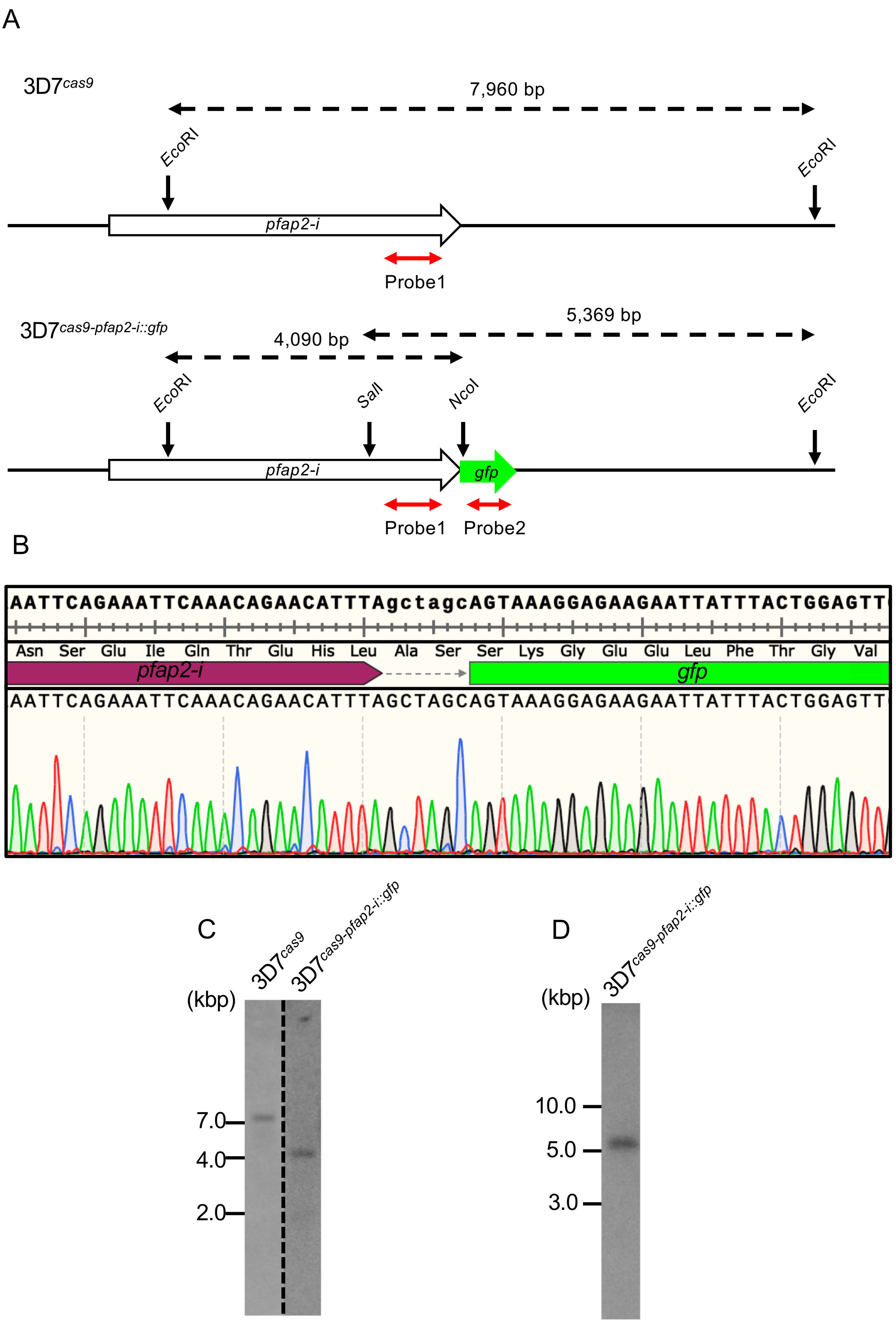

Figure S3. (A) Restriction maps of *pfap2-i* locus of 3D7^{cas9} and 3D7$^{cas9\text{-}pfap2\text{-}i::gfp}$ parasites. Enzyme recognition sites were shown by arrows. DNA regions indicated by red arrows were used for the Southern analyses in Fig. S3C and D. (B) Sequence analysis of the C-terminus of PfAP2-I of 3D7$^{cas9\text{-}pfap2\text{-}i::gfp}$. (C) Genomic DNA were purified from 3D7$^{cas9\text{-}pfap2\text{-}i::gfp}$ and then digested with *Eco*RI and *Nco*I. Southern hybridization was performed using the Probe DNA1, which were indicated by the red arrows in (A). Full length blot images are included in Fig. S4E. (D) Purified genomic DNA was also digested with *Eco*RI and *Sal*I. The digested fragment including *gfp* incorporated by HDR was detected by hybridizing with the Probe DNA2. Full length blot image is included in Fig. S4F.

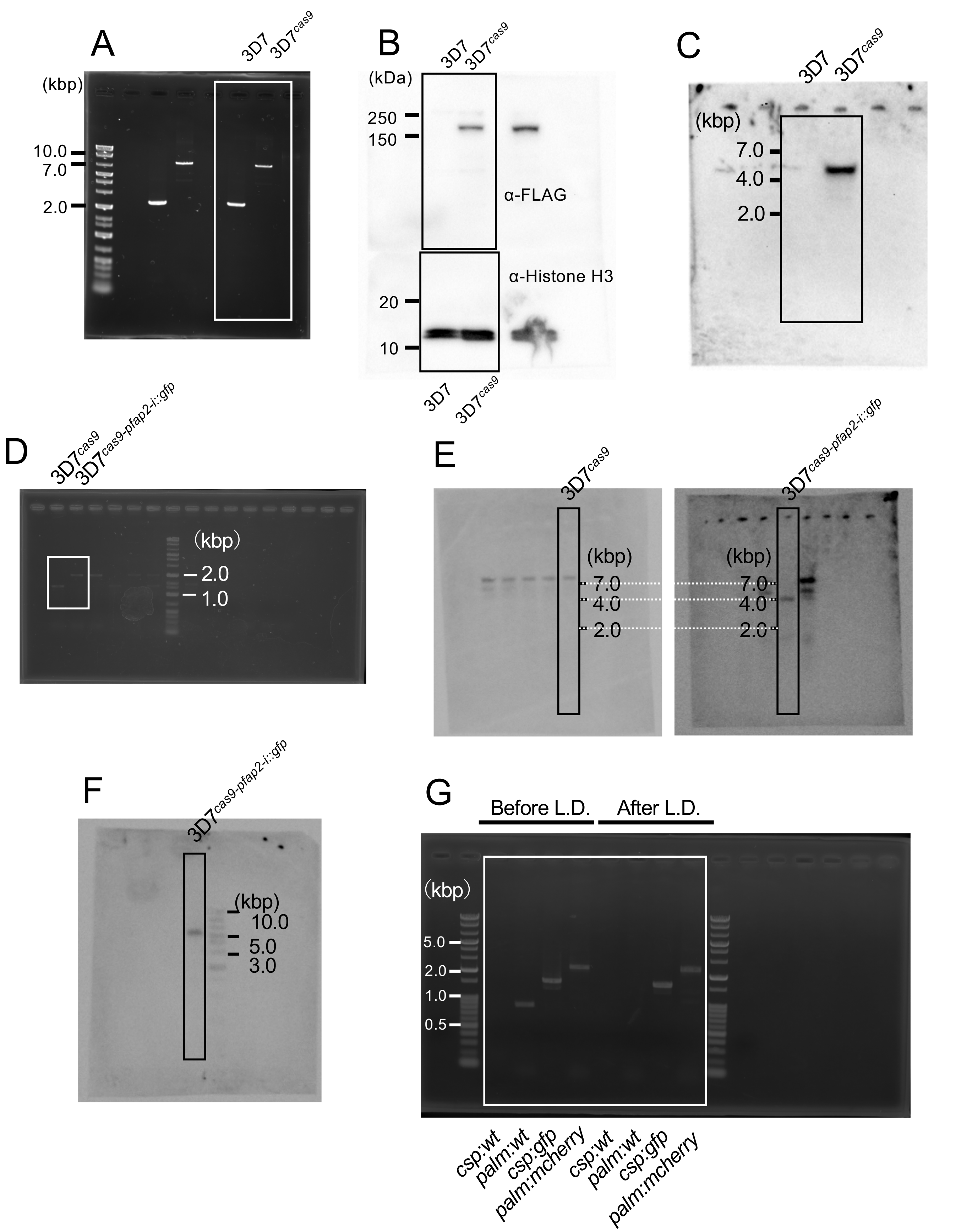

Figure S4: The original images of gel/blots used for figures. (A) Full length gel image of the genotyping PCR of the 3D7^{cas9} corresponding for Fig. 2B. (B) Full length western blot using anti-FLAG antibody for the 3D7^{cas9} corresponding Fig. 2C. (C) Full length image for Southern hybridization analysis of the 3D7^{cas9} corresponding Fig. S1. (D) Full length image of the genotyping PCR of the 3D7$^{cas9\text{-}pfap2\text{-}i::gfp}$ used in Fig. 3F. (E) Full length images for Southern hybridization analysis of the 3D7$^{cas9\text{-}pfap2\text{-}i::gfp}$ corresponding Fig. S3C. Sizes of signals between the 3D7^{cas9} and the 3D7$^{cas9\text{-}pfap2\text{-}i::gfp}$ was compared using molecular marker. (F) Full length image of Southern hybridization analysis of the 3D7$^{cas9\text{-}pfap2\text{-}i::gfp}$ using thee gfp gene as the probe DNA. This image was used in Fig. S3D. (G) Full length image of genotyping PCR of the 3D7$^{cas9\text{-}Pfg_red/green}$ corresponding Fig. 4B.
